# Blockade of the Hedgehog pathway downregulates estrogen receptor alpha signaling in breast cancer cells

**DOI:** 10.18632/oncotarget.12259

**Published:** 2016-09-26

**Authors:** Yumei Diao, Ani Azatyan, Mohammed Ferdous-Ur Rahman, Chunyan Zhao, Jian Zhu, Karin Dahlman-Wright, Peter G. Zaphiropoulos

**Affiliations:** ^1^ Department of Biosciences and Nutrition, Karolinska Institutet, Huddinge, Sweden

**Keywords:** glioma associated oncogene, GLI1, tamoxifen, drug targeting, GANT61

## Abstract

Anti-estrogen treatment, exemplified by tamoxifen, is a well-established adjuvant therapy for estrogen receptor alpha (ERα)-positive breast cancer. However, the effectiveness of this drug is limited due to the development of resistance. The Hedgehog (HH) signaling pathway is critical in embryonic development, and aberrant activation of this transduction cascade is linked to various malignancies. However, it remains unclear whether HH signaling is activated in human breast cancer and related to tamoxifen resistance. Deciphering how this pathway may be involved in breast cancer is a crucial step towards the establishment of targeted combinatorial treatments for this disease. Here, we show that the expression of the HH signaling effector protein GLI1 is higher in tamoxifen resistant compared to sensitive cells. Tamoxifen resistant cells have stronger ERα transcriptional activity relative to sensitive cells, even though the ERα expression is similar in both cell types. Knockdown of GLI1 attenuates cell proliferation and reduces ERα transcriptional activity in both sensitive and resistant cells, irrespective of estrogen stimulation. Combinatorial treatment of tamoxifen and the GLI antagonist GANT61 further suppresses the growth of sensitive and resistant cells relative to administration of only tamoxifen, and this was irrespective of estrogen stimulation. Moreover, a positive correlation between GLI1 and ERα expression was identified in breast cancer samples. Additionally, high GLI1 expression predicted worse distant metastasis-free survival in breast cancer patients. These data suggest that the HH pathway may be a new candidate for therapeutic targeting and prognosis in ERα-positive breast cancer.

## INTRODUCTION

Breast cancer is the most common cancer in women, affecting 12% of females worldwide [1], and is the leading cause of cancer related deaths in women [2]. Breast cancer most frequently originates from the lobe or the milk duct, both of which highly express estrogen receptor alpha (ERα). Thus, the majority of breast cancers are ERα-positive, which makes them suitable for selective ERα modulators, such as tamoxifen. Importantly, in 30% to 40% of patients receiving adjuvant tamoxifen therapy the tumor eventually relapses, and this is a significant clinical problem [3]. Clearly, additional and/or complementary approaches are necessary to more accurately define patients who will benefit from the above therapy and to design novel treatment strategies. Interestingly, recent work implicates that activation of Hedgehog (HH) signaling may have a role in the development of tamoxifen resistance in breast cancer [4].

The HH signaling pathway has critical roles in embryonic development and tumorigenesis [5–7]. Aberrant activation of HH signaling is involved in several types of malignant tumors [8], including basal cell carcinoma, medulloblastoma, rhabdomyosarcoma and cancers of the pancreas, colon, stomach, lung and prostate. The pathway is initiated by HH ligand [Sonic HH (SHH), Indian HH (IHH), Desert HH (DHH)] [7, 9–11] binding to Patched (PTCH1, PTCH2), a twelve trans-membrane domain protein. In the absence of HH ligands, PTCH inhibits the signaling of the seven trans-membrane domain protein and proto-oncogene Smoothened (SMO). Upon HH ligand binding, the PTCH inhibition of SMO is released and the signal is transduced to the terminal effectors, the GLI, Glioma associated oncogene, proteins (GLI1, GLI2, GLI3) [11]. GLI1 is a transcription factor that acts not only as a signaling effector but also represents a pathway target gene [12], amplifying the HH signal. Its expression levels thus correlate directly with pathway activity [13]. GLI1 is known to function as an oncogene [14], promoting cell proliferation and angiogenesis [15].

Possible links between HH signaling activation and the development of breast cancer have been widely studied. An elevated expression of HH ligands was associated with a basal-like breast cancer phenotype and a poor prognosis [16]. This may be the result of hypomethylation of the SHH promoter [17]. Genetic alternations in components of the HH pathway, including loss of PTCH1 or GLI1 amplification, were suggested to result in breast cancer [18, 19]. Consistently, transgenic mice that conditionally expressed GLI1 in the mammary epithelium developed mammary tumors [20]. Constitutive activation of HH signaling in MMTV-SmoM2 transgenic mice caused alterations in mammary gland morphology, increased proliferation, and changed stem/progenitor cell numbers [21]. In ERα-positive breast cancer cells, estrogen was found to act via GLI1, promoting the development of cancer stem cells and epithelial to mesenchymal transition [22]. Interestingly, neuropilin 2 (NRP2) signaling increased GLI1 expression in breast tumor initiating cells, with GLI1 also inducing BMI-1, a key stem cell factor, and NRP2 expression, establishing an autocrine loop [23]. These studies provide insights into the mechanisms of HH signaling activation in the mammary gland and its possible role in breast tumorigenesis.

An additional connection between HH signaling and ERα-positive breast cancer was suggested in 2012, when Ramaswamy et al reported that the HH pathway can mediate tamoxifen resistance in breast cancer cells. In this work evidence was provided that the PI3K/Akt pathway activates HH signaling, bypassing the blockade of ERα signaling that was elicited by tamoxifen treatment [4].

Our current results indicate that GLI1 is expressed to a higher extent in tamoxifen resistant compared to sensitive breast cancer cells. Interestingly, we also find that depletion of GLI1 decreases ERα protein levels, with concomitant reduction of ERα signaling activity in both tamoxifen resistant and sensitive cells. Furthermore, GLI1 depletion enhances tamoxifen cytotoxicity in both resistant and sensitive cells. These observations indicate that GLI1 may have a role not only for tamoxifen resistance but can also modulate ERα signaling in both sensitive and resistant cells.

## RESULTS

### Hedgehog signaling activity in the tamoxifen resistant LCC2 and their parental, tamoxifen sensitive MCF7 cells

**Figure 1:**
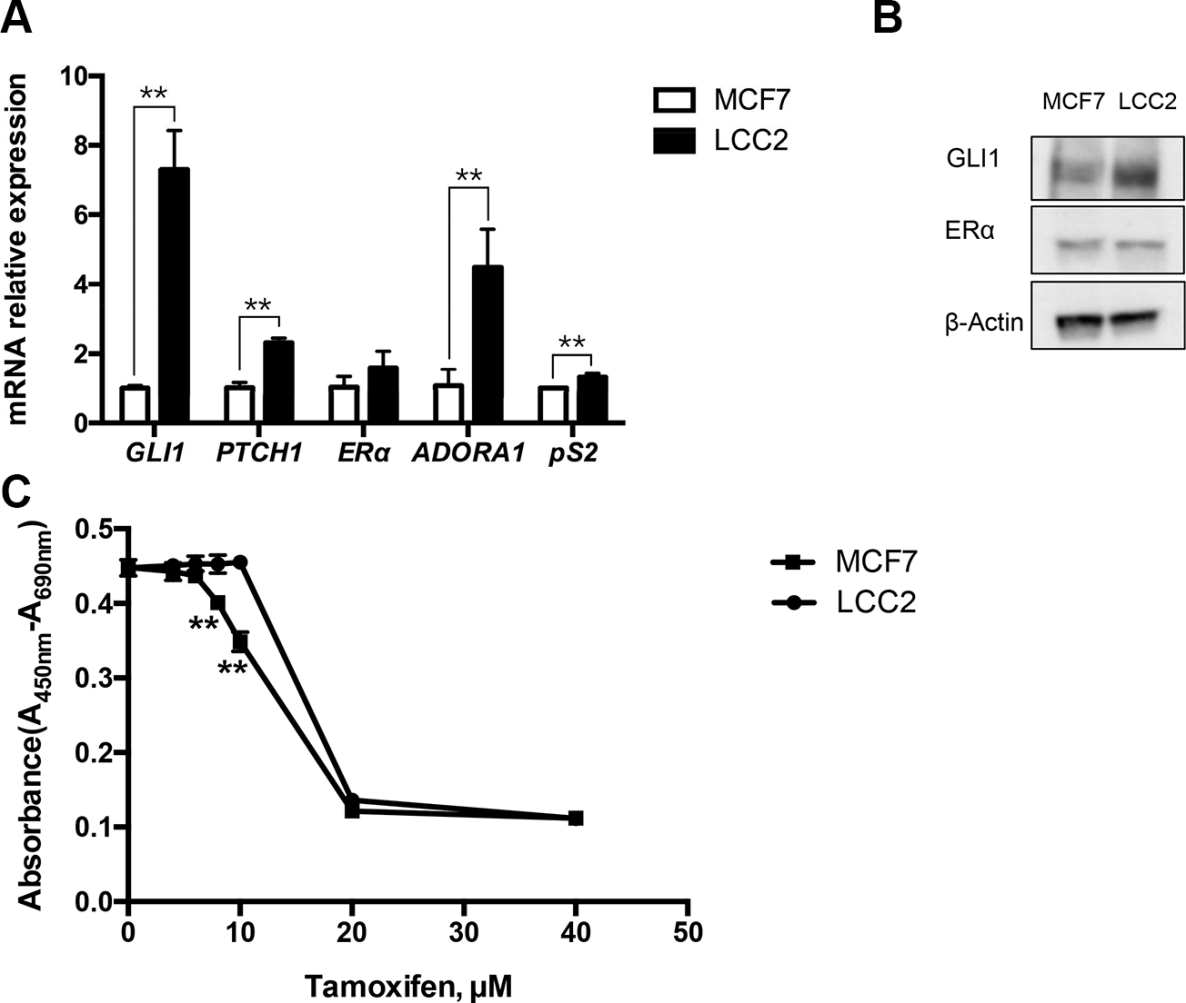
Characterization of tamoxifen sensitive MCF7 and tamoxifen resistant LCC2 breast cancer cells (**A**) Endogenous expression of *GLI1*, *PTCH1*, *ER*α, *ADORA1* and *pS2* in MCF7 and LCC2 cells was determined by real-time PCR. Data are represented as relative expression (2^−ΔΔCt^ values), calculated by subtracting the Ct value of the housekeeping gene *TBP* from the Ct value of the interrogated transcripts (ΔCt), and normalized to the ΔCt values obtained with MCF7. Representative data from one of three independent experiments are shown. Error bars indicate the standard deviation. **, Statistical significant, *P* < 0.01, compared to control, calculated by the Student's *t-test*. (**B**) Protein levels of GLI1, ERα and β-Actin in MCF7 and LCC2 cells were analyzed by Western blot. β-Actin was used as the endogenous protein control. (**C**) The effects of tamoxifen on cell viability. MCF7 and LCC2 cells were treated with 0, 4, 6, 8, 10, 20 or 40 μM tamoxifen and after 48 hours cell viability was determined with the WST-1 assay. The absorbance at 450 nm was measured with the reference wavelength set at 690 nm. Shown are data from triplicate measurements. Representative data from one of three independent experiments are shown. Error bars indicate the standard deviation. The two-way ANOVA analysis using Sidak's multiple comparisons was employed to calculate statistical significance (**P < 0.01).

Expression analysis of key markers of the activity of the HH signaling pathway i.e. GLI1 and PTCH1, revealed higher expression in the tamoxifen resistant LCC2 breast cancer cells compared to the parental, tamoxifen sensitive MCF7 cells (Figure 1A and 1B). Notably, MCF7 and LCC2 cells showed similar expression of the ERα mRNA and protein [24], however the ERα target genes *ADORA1* and *pS2* were upregulated in the resistant cells (Figure 1A and 1B). Cell viability assays indicated that LCC2 but not MCF7 cells are resistant to 10 μM tamoxifen, however 20 μM tamoxifen kills both cell types (Figure 1C).

This analysis demonstrates the higher HH signaling activity in the resistant cells and suggests that ERα activity may also be higher, despite the comparable ERα expression.

### Depletion of ERα or GLI1 reduces cell proliferation

**Figure 2:**
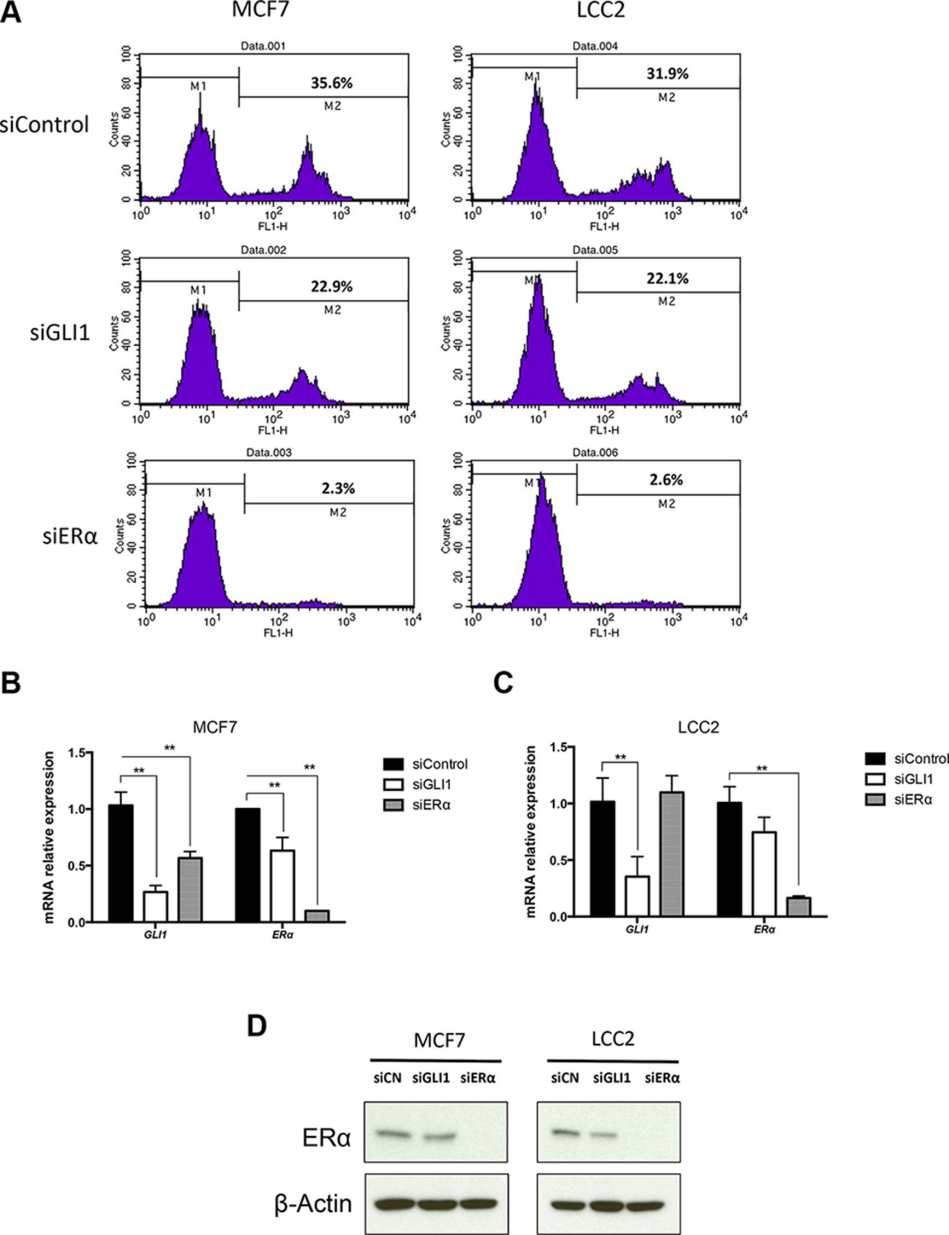
Depletion of GLI1 or ERα reduces the proliferation of MCF7 and LCC2 cells (**A**) MCF7 and LCC2 cells, cultured for 48 hours following transfection with control siRNA (siControl), GLI1 siRNA (siGLI1) or ERα siRNA (siERα), were subjected to the EdU incorporation assay for 1 hour. The percentage of cells labeled with Alexa Fluor 488 azide was detected by flow cytometry. The expression of *GLI1* and *ERα* in MCF7 (**B**) and LCC2 (**C**) cells, following siRNA knockdown of GLI1 or ERα, was determined by real-time PCR. Data are represented as relative expression (2-ΔΔCt values), calculated by subtracting the Ct value of the housekeeping gene *TBP* from the Ct value of the interrogated transcripts (ΔCt), and normalized to the ΔCt value obtained with control siRNA. Representative data from one of three independent experiments are shown. Error bars indicate the standard deviation. **, Statistical significant, *P* < 0.01, compared to control, calculated by the Student's *t-test*. (**D**) Protein levels of ERα in MCF7 and LCC2 cells, transfected with control siRNA (siCN), GLI1 siRNA (siGLI1) or ERα siRNA (siERα) for 48 hours, was determined by Western blot. β-Actin was used as the endogenous protein control.

To investigate the role of ERα and GLI1 in breast cancer cell proliferation, we transfected MCF7 and LCC2 cells with siRNAs targeting ERα or GLI1. RNA expression analysis showed that the ERα and GLI1 siRNAs successfully knocked down the respective genes in both cell lines (Figure 2B and 2C). Western blot analysis also showed ERα to be dramatically decreased by ERα siRNA treatment, and GLI1 to be downregulated by GLI1 siRNA treatment (Figure 2D, Supplementary Figure S1). Depletion of ERα resulted in a major reduction of the cell proliferation in both cell lines (Figure 2A), highlighting their dependence on ERα. Depletion of GLI1 also reduced the cell proliferation of the two cell lines, but to a lesser extent (Figure 2A).

These observations are in-line with the significance of ERα in breast cancer cells [3, 25, 26]. Moreover, they indicate that GLI1 can modulate proliferation not only in tamoxifen resistant but also in tamoxifen sensitive cells.

### GLI1 depletion reduces ERα activity assayed through an Estrogen Response Element (ERE) reporter

**Figure 3:**
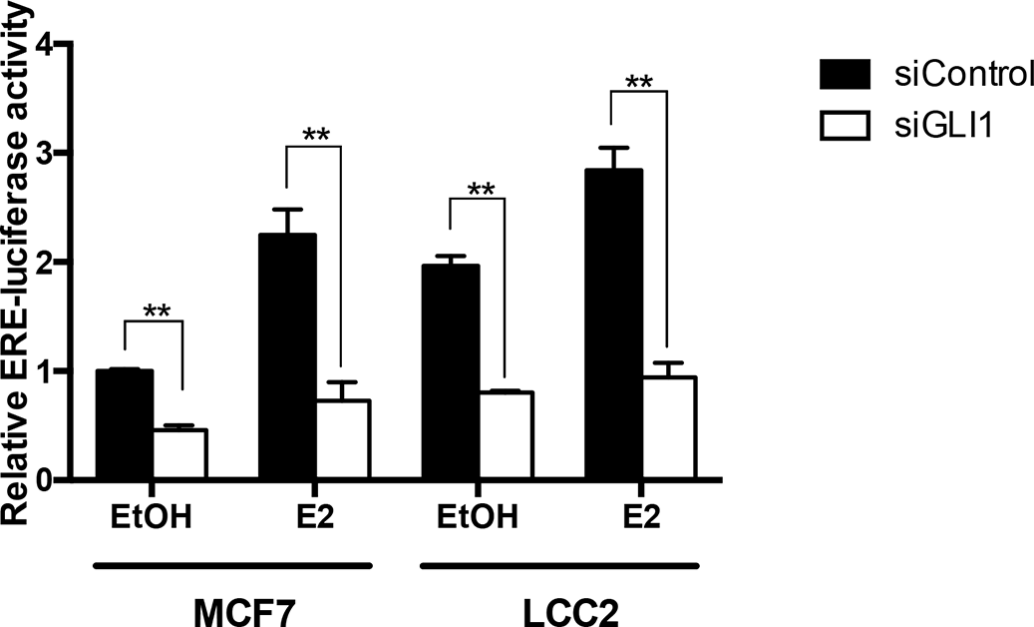
GLI1 depletion reduces the activity of an ERα reporter MCF7 and LCC2 cells were transfected with control siRNA (siControl) or GLI1 siRNA (siGLI1) and after 24 hours were co-transfected with the reporter plasmid ERE-TK-Luc and the pRL-TK control plasmid. Subsequently, both cell lines were treated with 10 nM E2 or ethanol (EtOH) for 24 hours in serum-deprived medium before harvesting. Luciferase expression was measured 48 hours after plasmid transfection. Shown are data from two independent experiments. Error bars indicate the standard error of the mean (SEM). **, Statistical significant, *P* < 0.01, compared to control, calculated by the Student's *t-test*.

To determine whether endogenous GLI1 expression may have an impact on ERα transcriptional activity, we used an Estrogen Response Element (ERE) luciferase reporter. GLI1 depletion reduced ERα activity both in MCF7 and LCC2 cells, irrespective of the presence or absence of estrogen (Figure 3, Supplementary Figure S2). Importantly, the basal level of the ERα transcriptional activity was higher in LCC2 compared to MCF7 cells, an observation in-line with the expression pattern of the ERα target genes *ADORA1* and *pS2* (Figure 1A).

These findings suggest an interplay of GLI1 with ERα signaling in both tamoxifen resistant and sensitive cells.

### GLI1 depletion decreases the expression of ERα and its target genes

**Figure 4:**
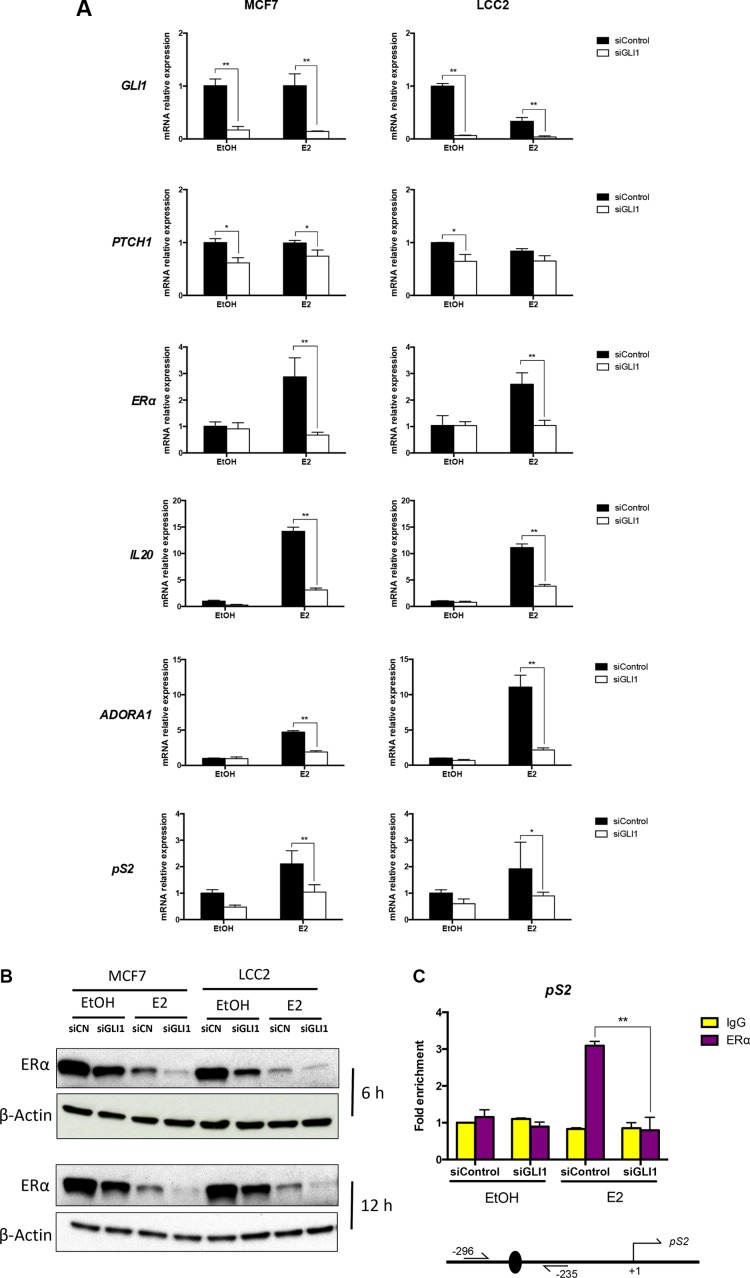
GLI1 depletion reduces the expression of ERα, its target genes and the binding to its targets (**A**) The expression of *GLI1*, *PTCH1*, *ERα* and its target genes, *IL20*, *ADORA1* and *pS2* in MCF7 and LCC2 cells treated with 10 nM E2 or ethanol (EtOH) for 3 hours in serum-deprived medium, following siRNA knockdown of GLI1, was determined by real-time PCR. Data are represented as relative expression (2^−ΔΔCt^ values), calculated by subtracting the Ct value of the housekeeping gene *TBP* from the Ct value of the interrogated transcripts (ΔCt), and normalized to the ΔCt value obtained with control siRNA in MCF7/LCC2 cells. Representative data from one of three independent experiments are shown. Error bars indicate the standard deviation. * or **, Statistical significant, *P* < 0.05 or *P* < 0.01 respectively, compared to control siRNA, calculated by the Student's *t-test*. (**B**) Protein levels of ERα and β-Actin in MCF7 and LCC2 cells treated with 10 nM E2 or ethanol (EtOH) for 6 and 12 hours in serum-deprived medium, 24 hours after transfection with control siRNA (siCN) or GLI1 siRNA (siGLI1), was determined by Western blot. β-Actin was used as the endogenous protein control. (**C**) Recruitment of ERα to the promoter of the *pS2* gene is diminished following GLI1 depletion. MCF7 cells were transfected with control siRNA or GLI1 siRNA and after 48 hours treated with 10 nM E2 or ethanol (EtOH) for 30 min in serum-deprived medium before harvesting and subjected to ChIP-qPCR analysis with an ERα antibody and PCR primers spanning the ERα binding site at the *pS2* gene promoter. The data presented are normalized to input DNA and expressed as fold enrichment over IgG. Shown are data from two independent experiments. Error bars indicate the standard error of the mean (SEM). **, Statistical significant, *P* < 0.01, compared to IgG control, calculated by the Student's *t-test*. A schematic diagram of the promoter region of the *pS2* gene, with the transcriptional start sites (+1) indicated by an arrow, the ERα binding site by a black oval and the position of the primers (−296, −235) is also shown. Note the increased ERα binding at the promoter following E2 treatment, which is eliminated by GLI1 depletion.

To address the functional consequences of the suggested GLI1 and ERα interplay, RNA expression analysis was used following GLI1 knockdown. GLI1 depletion was first confirmed and also shown to decrease the expression of the GLI1 target gene *PTCH1*. Moreover, the expression of *ERα* and its target genes *IL20*, *ADORA1* and *pS2* were also reduced in the context of estrogen treatment, while limited effects were observed without addition of estrogen (Figure 4A). The same assay was also performed using two additional ERα-positive breast cancer cell lines, ZR751 and T47D, resulting in a similar downregulation of *ERα*, *IL20* and *pS2* by GLI1 knockdown (Supplementary Figure S3A). Western blot analysis demonstrated that GLI1 depletion downregulated ERα in both MCF7 and LCC2 cells, irrespective of the absence or presence of estrogen for 6 or 12 hours (Figure 4B, Supplementary Figure S1). As noted before, estrogen treatment reduced ERα protein expression [24]. Consistently, ChIP analysis revealed decreased ERα binding at the promoter region of its target gene *pS2* [27–29] following GLI1 depletion in the presence of estrogen (Figure 4C).

### The GLI inhibitor GANT61 increases the cytotoxicity of tamoxifen on MCF7 and LCC2 cells, with or without addition of estrogen

**Figure 5:**
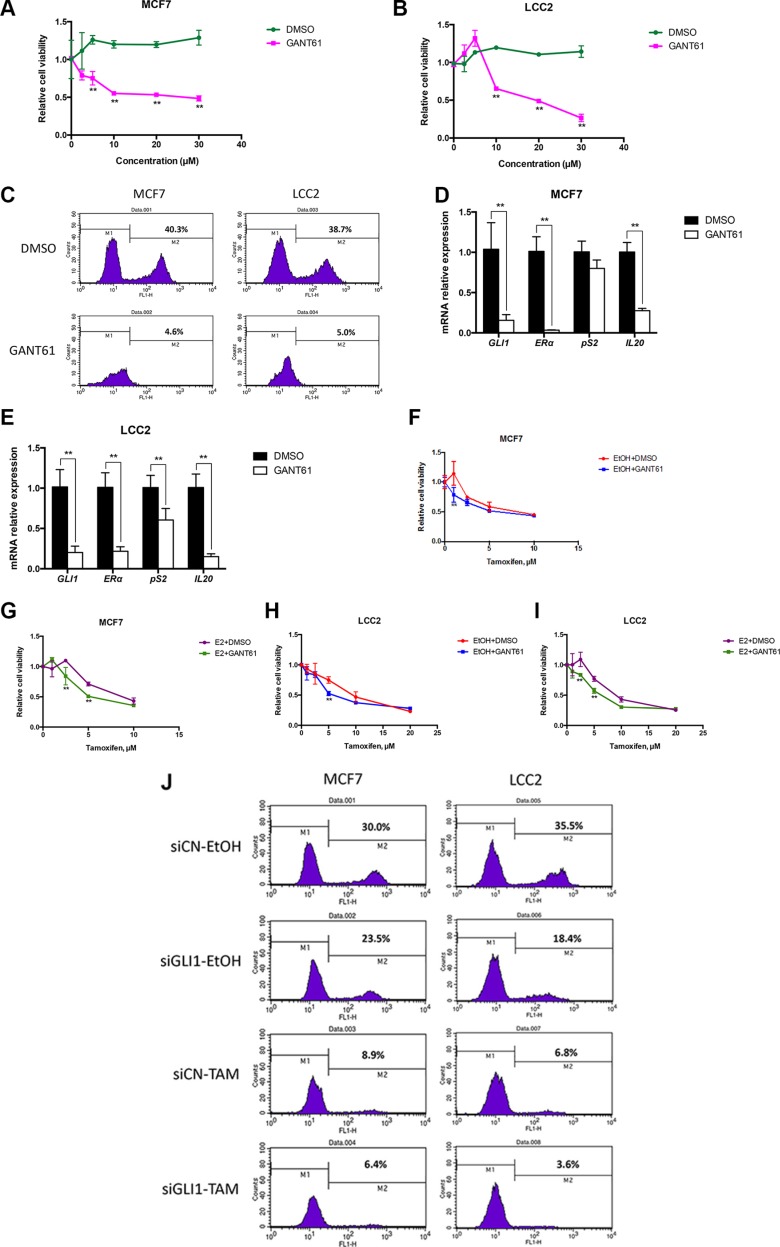
GANT61 increases tamoxifen cytotoxicity, irrespective of the presence or absence of estrogen (**A**), (**B**) GANT61 suppresses the cell viability of MCF7 and LCC2 cells. Both cell lines were treated with 0, 2.5, 5, 10, 20 and 30 μM GANT61 or DMSO as a control. After 48 hours cell viability was determined with the WST-1 assay. Error bars indicate the standard deviation. The two-way ANOVA analysis using Sidak's multiple comparisons was employed to calculate statistical significance (**P < 0.01). (**C**) GANT61 treatment reduces the cell proliferation of MCF7 and LCC2 cells. Both cell lines were treated with 10 μM GANT61 or DMSO as a control. After 48 hours cell proliferation was determined by the EdU incorporation assay using flow cytometry. (**D**), (**E**) The expression of *GLI1*, *ER*α, *pS2* and *IL20* in MCF7 and LCC2 cells treated with GANT61 or DMSO for 48 hours were measured by real-time PCR. Error bars indicate the standard deviation. **, Statistical significant, *P* < 0.01 respectively, compared to the DMSO control, calculated by the Student's *t-test*. (**F**–**I**) MCF7 and LCC2 cells were treated with 10 nM E2 or EtOH and 10 μM GANT61 or DMSO in the presence of different concentrations (0, 1, 2.5, 5, 10 or 20 μM) of tamoxifen, for 48 hours and the number of viable cells was measured with the WST-1 assay using a TECAN plate spectrophotometer. Shown are data from triplicate measurements expressed as percentage of control. Representative data from one of three independent experiments are shown. Error bars indicate the standard deviation. The two-way ANOVA analysis using Sidak's multiple comparisons was employed to calculate statistical significance (**P < 0.01). (**J**) MCF7 and LCC2 cells, cultured for 24 hours following transfection with control siRNA (siCN) or GLI1 siRNA (siGLI1) and treated with 10 μM tamoxifen (TAM) or ethanol (EtOH) for 48 hours, were subjected to the EdU incorporation assay for 1 hour. The percentage of cells labeled with Alexa Fluor 488 azide was detected by flow cytometry.

To examine possible therapeutic applications of the HH signaling interplay with ERα, we investigated whether treatment of MCF7 and LCC2 cells with the GLI inhibitor GANT61 [30] may enhance tamoxifen cytotoxicity. First, we tested the effects of only GANT61 administration on cell viability and cell proliferation. As expected, GANT61 treatment resulted in a dose-dependent reduction of the viability of MCF7 and LCC2 cells (Figure 5A and 5B). Additionally, the proliferation of both cell lines was inhibited (Figure 5C) and the mRNA expression of ERα and its corresponding target genes were downregulated by 48-hour GANT61 treatment (Figure 5D and 5E). Interestingly, a 24-hour GANT61 treatment also had an obvious impact on cell proliferation (Supplementary Figure S4A) and mRNA expression (Supplementary Figure S4B).

Moreover, GANT61 co-administration with tamoxifen further decreased the cell growth of MCF7 and LCC2 cells, and this was irrespective of the presence or absence of estrogen (Figure 5F–5I). SiRNA depletion of GLI1 also enhanced the impact of tamoxifen in reducing the proliferation of the two cell lines (Figure 5J). Similar enhancement of the tamoxifen impact by GLI1 depletion was also observed in ZR751 and T47D cells (Supplementary Figure S3B). However, in ZR751 cells GLI1 depletion reduced cell proliferation to a comparable extent as tamoxifen treatment, suggesting an increased significance of GLI1 in this cellular context.

Thus, the role of GLI1 for the proliferation of ERα-positive breast cancer cells may be exploited for therapeutic purposes, and drug targeting of GLI1 could enhance the tamoxifen efficacy in the treatment of breast cancer.

### Correlation between GLI1 and ERα/ERα target gene expression in breast cancer - Impact of GLI1 expression in distant metastasis-free survival

**Figure 6:**
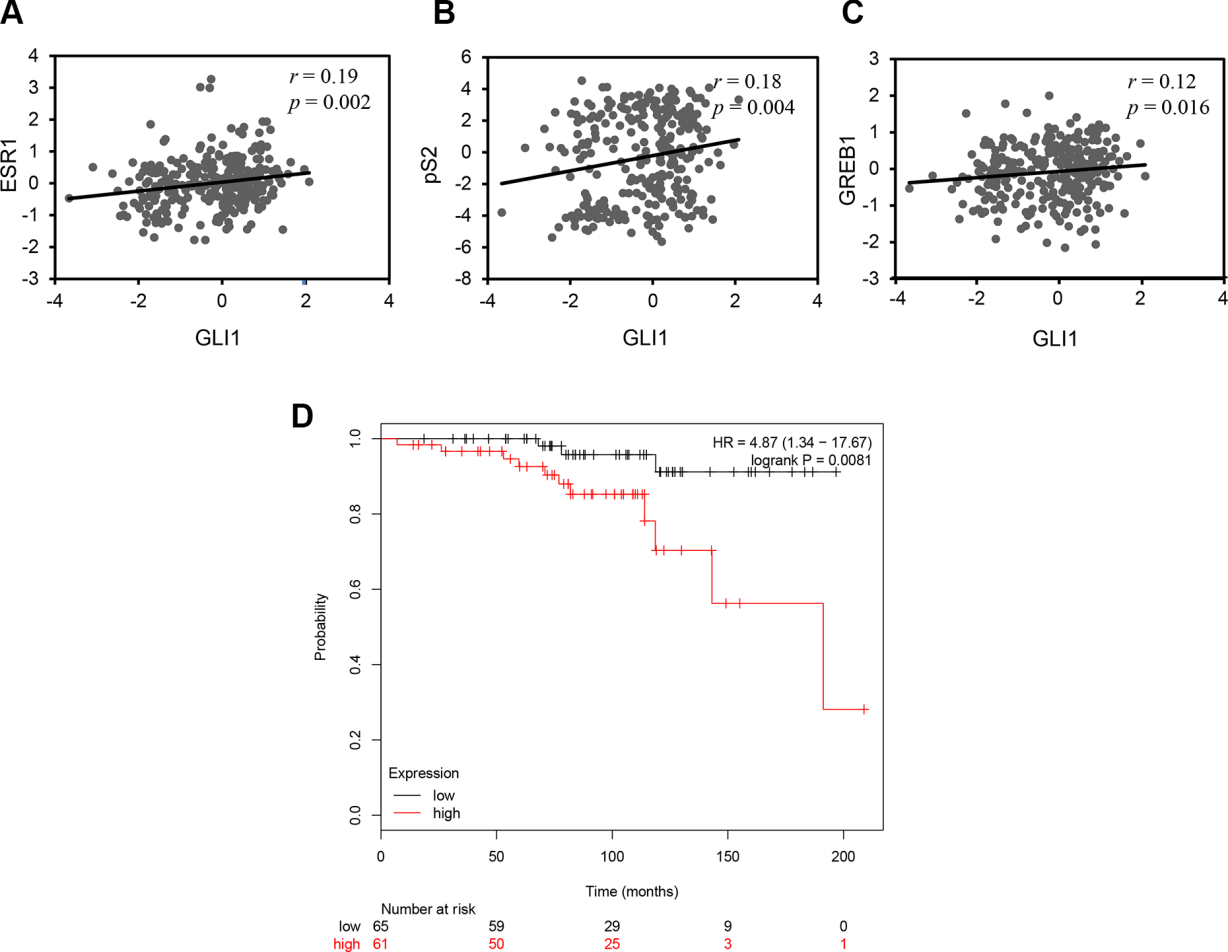
*GLI1* expression positively correlates with the expression of *ESR1* and its target genes and is a negative prognostic marker in breast cancer (**A**–**C**) Scatter plots showing significant correlation between *GLI1* and *ESR1* (A), *GLI1* and *pS2* (B), *GLI1* and *GREB1* (C) expression in a publically available breast cancer dataset, test statistics were from Pearson product-moment correlation. (**D**) Kaplan-Meier plot showing that high *GLI1* expression correlates with worse distant metastasis-free survival (DMFS) in patients with ERα-positive, Grade 1 breast cancer. The Kaplan-Meier plot is stratified for high (red) and low (black) *GLI1* expression (*n* = 126; *P* = 0.0081).

To explore the clinical relevance of the effect of GLI1 on ERα signaling and breast cancer, we examined the expression of *GLI1, ESR1* (the gene encoding ERα) and known ERα target genes in a dataset of breast cancer samples from 286 individuals [31]. Our analysis revealed that the expression of *GLI1* positively correlates with *ESR1* (Figure 6A) and the ERα targets genes *pS2* (Figure 6B) and *GREB1* (Figure 6C). Then we examined the prognostic role of GLI1 expression for breast cancer patients using the Kaplan-Meier Plotter dataset [32]. We observed that high GLI1 expression is associated with poor distant metastasis-free survival (DMFS) in 126 patients with Grade 1, ERα-positive breast cancer (Figure 6D).

These findings suggest that GLI1 may represent not only a therapeutic target but could also be a valuable prognostic marker for breast cancer patients.

## DISCUSSION

Our data indicate that GLI1 depletion reduces the proliferation of both tamoxifen resistant and sensitive breast cancer cells (Figure 2). Moreover, the GLI inhibitor GANT61 increases the cytotoxicity of tamoxifen on both resistant and sensitive cells and this is irrespective of the activation of ERα signaling by estrogen (Figure 5F–5I). Additionally, GLI1 knockdown enhanced the effect of tamoxifen in reducing the proliferation of four breast cancer cell lines (Figure 5I, Supplementary Figure S3B). These data contrast earlier observations on tamoxifen and cyclopamine, a HH signaling inhibitor acting upstream of the GLI factors, co-treatments, which indicated that the two drugs counter each other's effect [33]. Interestingly, the GLI1 depletion confers a reduction of ERα transcriptional activity in the LCC2 and MCF7 cells, with or without estrogen treatment (Figure 3). Note that LCC2 cells are less sensitive than MCF7 to estrogen-mediated increase in proliferation [24]. Further analysis demonstrated that GLI1 depletion reduces ERα protein expression (Figure 4B). Additionally, in the context of ERα activation, GLI1 depletion elicited a consistent reduction in the mRNA expression of ERα and the ERα target genes analyzed (Figure 4A). These findings may suggest that GLI1, acting as transcription factor, regulates ERα expression when ERα signaling is on. However, exogenous expression of GLI1 did not increase the mRNA and protein levels of ERα (Supplementary Figure S5A and S5B), implying that the impact of GLI1 on ERα is more complicated than a typical direct GLI1 target, e.g. PTCH1 (Supplementary Figure S5A). To further test the hypothesis of transcriptional regulation of ERα by GLI1 we applied ConSite [34], a web-based tool for binding sites prediction, and took advantage of the Position Specific Frequency Matrices (PSFM) of the GLI1 binding sites [35]. Combined with the predicted GLI1 binding consensus sequence [36] and following BLAST analysis with the estrogen receptor 1 gene (*ERS1*), three common hits were identified. However, we were unable to detect convincing GLI1 binding on the *ERS1* gene promoter, possibly reflecting limitations of ChIP validated GLI1 antibodies. Unsuccessful were also our efforts to detect possible GLI1/ERα protein interactions using immunoprecipitations assays.

The mechanisms of tamoxifen resistance are not entirely clear, although this phenomenon is commonly observed. Tamoxifen resistance may be mediated by altered ERα function/ERα signaling. Loss of ERα function [37–39] and ERα mutations [40–42] are well-studied mechanisms that induce resistance to tamoxifen. Moreover, the complexity of ERα signaling is indicative of additional pathways that may be involved in tamoxifen resistance [43]. These include PI3K (phosphoinositide 3-kinase) [4], HER2 (human epidermal growth factor receptor 2) [44], MAPK (mitogen-activated protein kinase) [45], and NOTCH [5, 46]. We specifically examined the activity of PI3K/Akt signaling in MCF7 and LCC2 cells. The basal level of phosphorylated Akt (p-Akt) was found to be comparable. Moreover, PI3K/Akt signaling in both cell lines could be activated by insulin treatment (Supplementary Figure S6.) Although Ramaswamy et al [4] showed that treatment with the PI3K inhibitor LY294002 elicited a 50% decrease in GLI-dependent luciferase activity in tamoxifen resistant but not in tamoxifen sensitive MCF7 cells, they did not directly demonstate that the PI3K/Akt signaling pathway is more active in the tamoxifen resistant cells.

There is strong evidence that the ability of tamoxifen to function as an ERα agonist or antagonist is dependent on whether it recruits co-activators or co-repressors to the ERα transcription complex [47–50]. This may partly explain the observation of a tamoxifen-induced increase in the proliferation of the ERα-positive ZR751 and BT474 breast cancer cells, which moreover, was accompanied by a sustained upregulation of GLI1 expression [51].

Resistance to tamoxifen is a major therapeutic concern for the treatment of breast cancer. The clinical and experimental evidence on both intrinsic and acquired resistance are well documented in many reviews [52–54]. Changes in the expression of ERα, ERα pathway components or in signaling cascades interacting with ERα are observed in experimental models of tamoxifen resistance. Some findings are consistent with the clinical data, while others are not. To create effective therapeutic approaches, a further focus on the tumor itself and the detailed classification of breast tumor subtypes, with personalized treatment options should be considered. Due to the complexity of tumor heterogeneity and tumor environment, emerging high throughput technologies will be indispensable to study and potentially overcome tamoxifen resistance.

Our data highlighting that the expression of *GLI1* correlates with *ESR1*, *pS2* and *GREB1* using a publicly available dataset (Figure 6A, 6B and 6C), suggest that *GLI1* may represent a gene with implications in breast cancer. Indeed, we observe that high GLI1 expression predicts worse DMFS in Grade 1, ERα-positive breast cancer patients (Figure 6D). It would be interesting but also challenging to verify GLI1 as prognostic marker in a larger number of patients and/or other subtypes of breast cancer.

Taken together, in this work we have demonstrated that tamoxifen cytotoxicity can be enhanced by blockade of the HH pathway, reflecting a cross-talk between ERα and GLI1 signaling, both in tamoxifen resistant and sensitive breast cancer cells. Therefore GLI1 may be a potential therapeutic target and moreover, could also act as a prognostic marker in breast cancer.

## MATERIALS AND METHODS

### Cell culture

Tamoxifen resistant cell line LCC2 and its parental, tamoxifen sensitive cell line MCF7 were kind gifts of Staffan Strömblad (Karolinska Institutet, Stockholm, Sweden). Both cell lines were cultured in DMEM high glucose medium with 10% fetal calf serum (FCS) supplemented with 100 IU/ml penicillin/streptomycin and maintained in a 5% CO_2_ humidified incubator. DMEM, penicillin/streptomycin, and trypsin were purchased from Sigma-Aldrich. 4-Hydroxytamoxifen (T176-10MG) and 17β-Estradiol (E2, E2758-250MG) were obtained from Sigma-Aldrich. For the experiments evaluating E2 or tamoxifen treatment, FCS with dextran-coated charcoal and DMEM without phenol red were used. Ethanol was the vehicle control.

### SiRNA transfection

Cells were transfected with 50 nM siRNA. GLI1 siRNAs and control siRNAs were purchased from Sigma-Aldrich, ERα siRNA (sc-44204) was purchased from Santa Cruz Biotechnology. Lipofectamine^®^ RNAiMAX Transfection Reagent (Invitrogen) was used according to the manufacturer's protocol.

### Cell proliferation

Cell proliferation assays were performed essentially as previously described [55]. Briefly, 5 × 10^5^ cells per well were seeded in 6-well plates, treated with siRNAs for 48 hours, followed by an 1 hour 10 μM EdU (5-ethynyl- 2′-deoxyuridine) incubation. EdU was detected by a fluorescent-azide coupling reaction (Click-iT, Invitrogen). For each treatment, 10 000 cells were analyzed on a FACS calibur machine (BD Biosciences, Stockholm, Sweden). Cell cycle distribution was calculated using the CellQuest software (BD Bioscience).

### Cell viability

Cells were seeded into 96-well plates 24 hours before starvation. GANT61 was dissolved in DMSO (dimethyl sulfoxide), whereas E2 in ethanol. Cells were treated with 10 nM E2 or EtOH and 10nM GANT61 or DMSO in the presence of different concentrations of tamoxifen, and then incubated with the indicated combination of drugs for 48 hours. Metabolic activity was measured with the WST-1 (Water Soluble Tetrazolium salt 1) cell proliferation reagent (Roche), and the number of viable cells was quantified at 450 nm using a TECAN plate spectrophotometer, with the reference wavelength set at 690 nm. Each measurement represents the mean of triplicates. The ordinary two-way ANOVA test was performed using the GraphPad Prism version 6.0d.

### RNA preparation, cDNA synthesis and real-time PCR

Total RNA was isolated with the RNeasy mini kit (Qiagen, Hamburg, Germany) according to the manufacturer's protocol. cDNA synthesis was performed with random N6 primers (New England Biolabs) and Superscript III (Invitrogen). Real-time PCR was carried out with the FastStart Universal SYBR Green Master (Rox) (Roche) on a 7500 fast real-time PCR system (Applied Biosystems), with primers designed to detect *GLI1*, *PTCH1*, *ER*α, *IL20, ADORA1, pS2* and *TBP* (Table 1). All amplifications were run at least in triplicate and the fold change was normalized to the expression of *TBP*. The relative expression was determined by the ΔCt method. All RNA expression experiments were done at least in triplicate and representative experiments are shown.

**Table 1:** Primer sequences for qRT-PCR analysis

TBP-E3 forward	5′ GCCAGCTTCGGAGAGTTCTGGGATT
TBP-E4 reverse	5′ CGGGCACGAAGTGCAATGGTCTTTA
GLI1-E11 forward	5′ CAGCTACATCAACTCCGGCCAATAGGG
GLI1-E12 reverse	5′ TGCTGCGGCGTTCAAGAGAGACTG
PTCH1-E17 forward	5′ AATGGGTCCACGACAAAGCCGACTA
PTCH1-E18 reverse	5′ TCCCGCAAGCCGTTGAGGTAGAAAG
ERα forward	5′ GCTACGAAGTGGGAATGATGAAAG
ERα reverse	5′ TCTGGCGCTTGTGTTTCAAC
IL20 forward	5′ CTGCCTCCTGCGCCATTTGC
IL20 reverse	5′ TCATGTGGGCATGACAGAGC
ADORA1 forward	5′ TTCCACACCTGCCTCATGGT
ADORA1 reverse	5′ GCGGTCCACAGCAATTGC
pS2 forward	5′ CATCGACGTCCCTCCAGAAGAG
pS2 reverse	5′ CTCTGGGACTAATCACCGTGCTG

### Western blot

For Western blot analysis, cells were lysed with RIPA buffer (150 mM NaCl, 50 mM Tris base pH 8.0, 1 mM EDTA, 0.5% sodium deoxycholate, 1% NP-40, 0.1% sodium dodecyl sulfate, 1 mM DTT, 1 mM PMSF, and 1 mM Na_3_VO_4_) supplemented with Complete Protease Inhibitor Tablets (Roche) and Phosphatase Inhibitor Cocktail 3 (Sigma). Proteins were separated on a 7.5% sodium dodecyl sulfate polyacrylamide gel electrophoresis (PAGE) followed by transfer (220 mA for 1 hour) to an Immobilon-P membrane (Millipore). The membrane was incubated at 4°C overnight in StartingBlock™ T20 (TBS) Blocking Buffer (#37543, Thermo Scientific) with monoclonal anti-mouse β-Actin antibody (A5441, Sigma-Aldrich), anti-rabbit GLI1 Ab (#2553, Cell Signaling Technology) or ERα antibody (sc-543, Santa Cruz Biotechnology), followed by incubation with goat anti-rabbit or anti-mouse secondary antibodies for 1 hour in StartingBlock™ T20 (TBS) Blocking Buffer and visualized using Pierce ECL chemiluminescent substrate (Thermo Scientific).

### Luciferase reporter assay

Cells were transfected with 50 nM GLI1 siRNAs or control siRNA. After 24 hours cells were co-transfected with the reporter plasmid ERE-TK-Luc and the pRL-TK control plasmid, which contains the Renilla luciferase gene, for normalizing transfection efficiency. Plasmid transfection was done using Lipofectamine 3000 (Invitrogen). After 24-hour plasmid transfection, the cells were changed to serum-deprived medium, incubated overnight, and then treated with 10 nM E2 or vehicle for 24 hours prior to harvesting. Luciferase activity was measured using the Dual-Luciferase Reporter Assay (Promega). The reporter plasmid ERE-TK-Luc has been described previously [56]. Multiple *t-test* analysis (corrected for multiple comparisons using the Holm-Sidak method) was performed with GraphPad Prism 6.0d.

### Chromatin immunoprecipitation (ChIP)

Cells were seeded in 150 mm dishes and were transfected with GLI1 siRNA or control siRNA for 48 hours. Cells were then treated with vehicle or 10 nM E2 for 30 min before harvesting and chromatin preparation. ChIP assays were performed essentially as described [57]. Briefly, 5 μg of ERα antibody (sc-543, Santa Cruz Biotechnology) or normal rabbit IgG (sc-2027, Santa Cruz Biotechnology) were conjugated to Dynabeads^®^ Protein A beads (Life Technologies), then antibody-bound beads were incubated with sonicated cell lysates. Immunoprecipitated DNA was purified using the QIAquick PCR Purification Kit and quantified by PCR. Input DNA was used to produce standard curves and the ChIP data were converted to percentages of total input. The PCR primer sequences are given in Table 2, with the negative control primer set (ERα ChIP NC) originating from the *ESR1* gene, 11 kb downstream of the transcription start site.

**Table 2:** Primer sequences for ChIP analysis

pS2 ChIP forward	5′ CCGGCCATCTCTCACTATGAA
pS2 ChIP reverse	5′ CCTCCCGCCAGGGTAAATAC
ERα ChIP NC forward	5′ CCTGATCTGGTTCTTCCTCTGCAT
ERα ChIP NC reverse	5′ CCAAATACAAGGGCTTGATTGCCA

### Statistics

Multiple *t*-test and ordinary two-way ANOVA test were performed using GraphPad Prism 6.0d. *P* values lower than 0.05 were considered as significant.

## SUPPLEMENTARY MATERIALS FIGURES


