# Supplementary material for: Blockade of the Hedgehog pathway downregulates estrogen receptor alpha signaling in breast cancer cells
Source: Oncotarget. 2016 Sep 26;7(44):71580–93. doi: 10.18632/oncotarget.12259 (PMC5342103; doi:10.18632/oncotarget.12259)
Supplement: Supplementary file 1 [file oncotarget-07-71580-s001.pdf]

Blockade of the Hedgehog pathway downregulates estrogen receptor alpha signaling in breast cancer cells

Supplementary Materials

Cell culture

The ERα-positive breast cancer cell lines ZR751 and T47D were purchased from ATCC. ZR751 cells were cultured in RPMI1640 medium with 10% fetal calf serum (FCS) supplemented with 1% L-glutamine, 10 mM HEPES, 1 mM Na-pyruvate. T47D cells were cultured in the same medium as ZR751 with the addition of 8 µg/ml insulin. 100 IU/ml penicillin/streptomycin was also added to the media and the cells maintained in a 5% CO_2 humidified incubator. RPMI1640, L-glutamine, HEPES, Na-pyruvate and insulin were purchased from Sigma-Aldrich. For the experiments evaluating E2 or tamoxifen treatment, FCS with dextran-coated charcoal and DMEM without phenol red were used. Ethanol was the vehicle control.

Plasmid transfection

Cells were seeded in 6-well plates and transfected with 2.5 µg GLI1 expression construct (FLAG-tagged GLI1) or pCMV5 vector per well. Lipofectamine® 3000 Transfection Reagent (Invitrogen) was used according to the manufacturer's protocol.

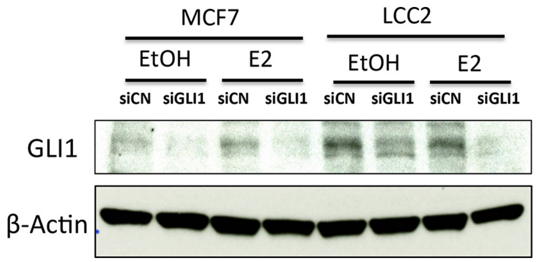

Supplementary Figure S1: The effectiveness of GLI1 depletion mediated by siRNAs. The protein levels of GLI1 in MCF7 and LCC2 cells treated with 10 nM E2 or ethanol (EtOH) in serum-deprived medium, 24 hours after transfection with control siRNA (siCN) or GLI1 siRNA (siGLI1), was determined by Western blot analysis. β-Actin was used as the endogenous protein control.

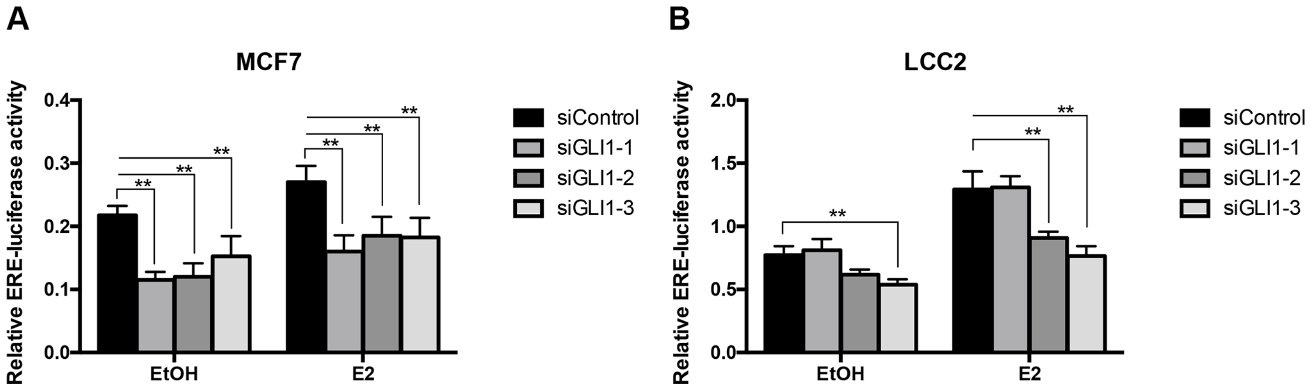

Supplementary Figure S2: The effectiveness of individual GLI1 siRNAs in reducing the activity of the ERα reporter. MCF7 (**A**) and LCC2 (**B**) cells were transfected with control siRNA (siControl) or three GLI1 siRNAs (siGLI1-1, siGLI1-2 to siGLI1-3). Following 24 hours after siRNA transfection, cells were co-transfected with the reporter plasmid ERE-TK-Luc and the control plasmid pRL-TK. Subsequently, both cell lines were treated with 10 nM E2 or ethanol (EtOH) for 24 hours in serum-deprived medium before harvesting. Luciferase expression was measured 48 hours after plasmid transfection. Error bars indicate the standard deviation. **, Statistical significant, $P < 0.01$, compared to control, calculated by the Student's t-test.

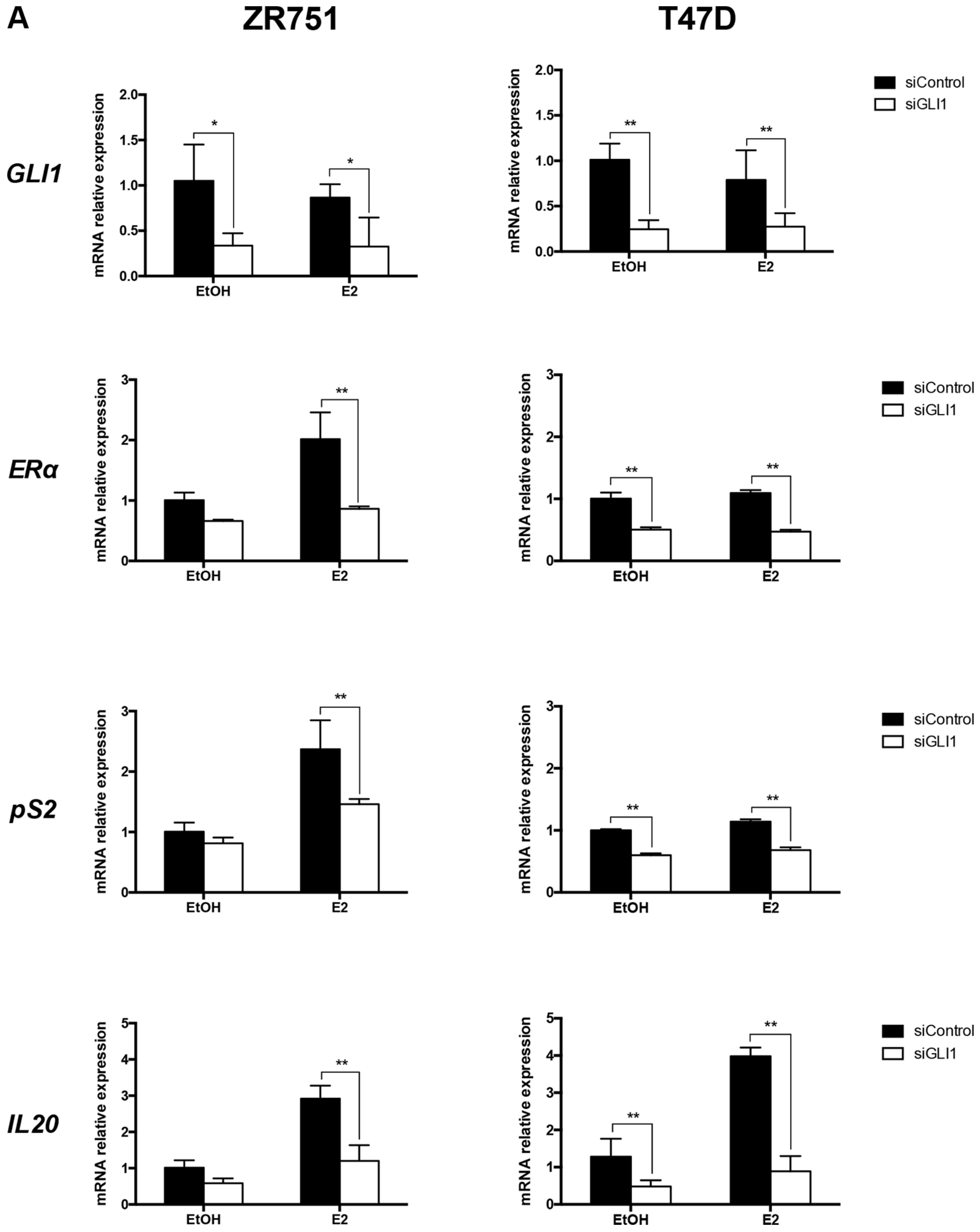
A
ZR751
T47D
GLI1
ERα
pS2
IL20
siControl
siGLI1
mRNA relative expression
EtOH
E2
*
**

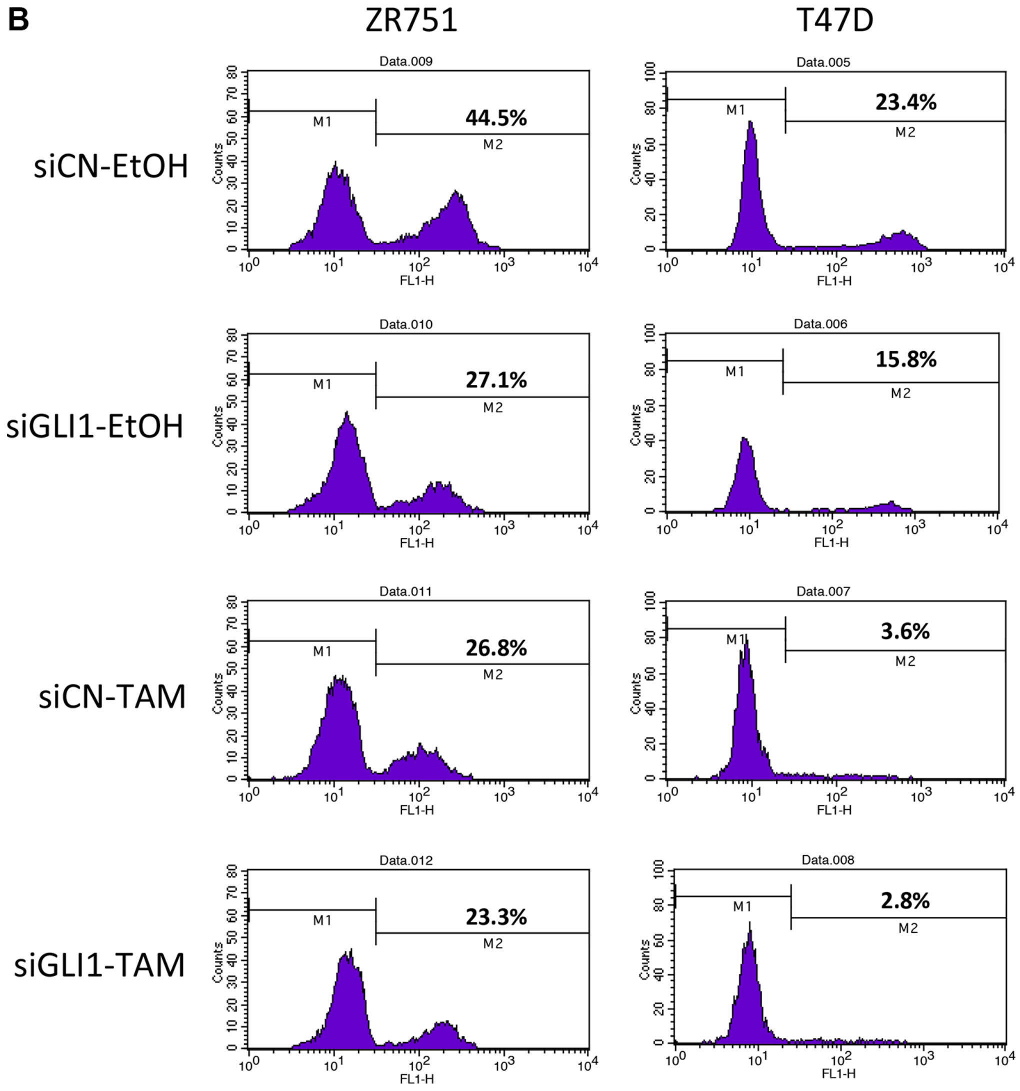

Supplementary Figure S3: GLI1 depletion reduces the expression of ERα and its target genes in ZR751 and T47D cells. (**A**) The expression of *GLI1*, *ERα, pS2* and *IL20* in ZR751 and T47D cells treated with 10 nM E2 or ethanol (EtOH) for 3 hours in serum-deprived medium, following siRNA knockdown of GLI1, was determined by real-time PCR. Data are represented as relative expression ($2^{-\Delta\Delta Ct}$ values), calculated by subtracting the Ct value of the housekeeping gene *TBP* from the Ct value of the interrogated transcripts (ΔCt), and normalized to the ΔCt value obtained with control siRNA in ZR751/T47D cells. Representative data from one of three independent experiments are shown. Error bars indicate the standard deviation. * or **, Statistical significant, $P < 0.05$ or $P < 0.01$ respectively, compared to control siRNA, calculated by the Student's *t*-test. (**B**) GLI1 depletion enhances the inhibition of cellular proliferation elicited by tamoxifen in ZR751 and T47D cells. ZR751 and T47D cells, cultured for 24 hours following transfection with control siRNA and GLI1 siRNA and treated with 10 μM tamoxifen (TAM) or ethanol (EtOH) for 48 hours, were subjected to the EdU incorporation assay for 1 hour. For both ZR751 and T47D cells the percentage of cells labeled with Alexa Fluor 488 azide was detected by flow cytometry.

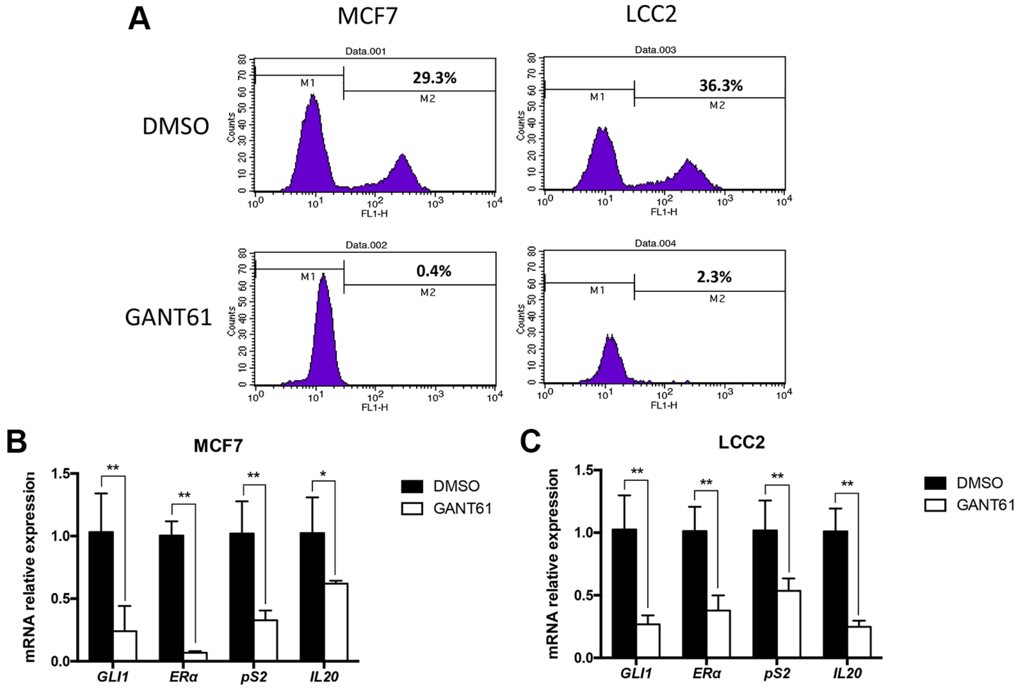

Supplementary Figure S4: GANT61 decreases cell proliferation and inhibits GLI1 and ERα/ERα target gene expression in MCF7 and LCC2 cells. (**A**) MCF7 and LCC2 cells, maintained in complete medium were treated with 10 µM GANT61 or DMSO as a control for 24 hours and subjected to the EdU incorporation assay for 1 hour. For both MCF7 and LCC2 the percentage of cells labeled with Alexa Fluor 488 azide was detected by flow cytometry. (**B**), (**C**) GANT61 reduces the mRNA expression of ERα and its target genes. The expression of *GLI1, ERα, pS2* and *IL20* in MCF7 (B) and LCC2 (C) cells, following 24-hour 10 µM DMSO or GANT61 treatment, was determined by real-time PCR. Data are represented as relative expression ($2^{-\Delta\Delta Ct}$ values), calculated by subtracting the Ct value of the housekeeping gene *TBP* from the Ct value of the interrogated transcripts (ΔCt), and normalized to the ΔCt value obtained with the DMSO control. Representative data from one of three independent experiments are shown. Error bars indicate the standard deviation. * or **, Statistical significant, $P < 0.05$ or $P < 0.01$, compared to control, calculated by the Student's *t*-test.

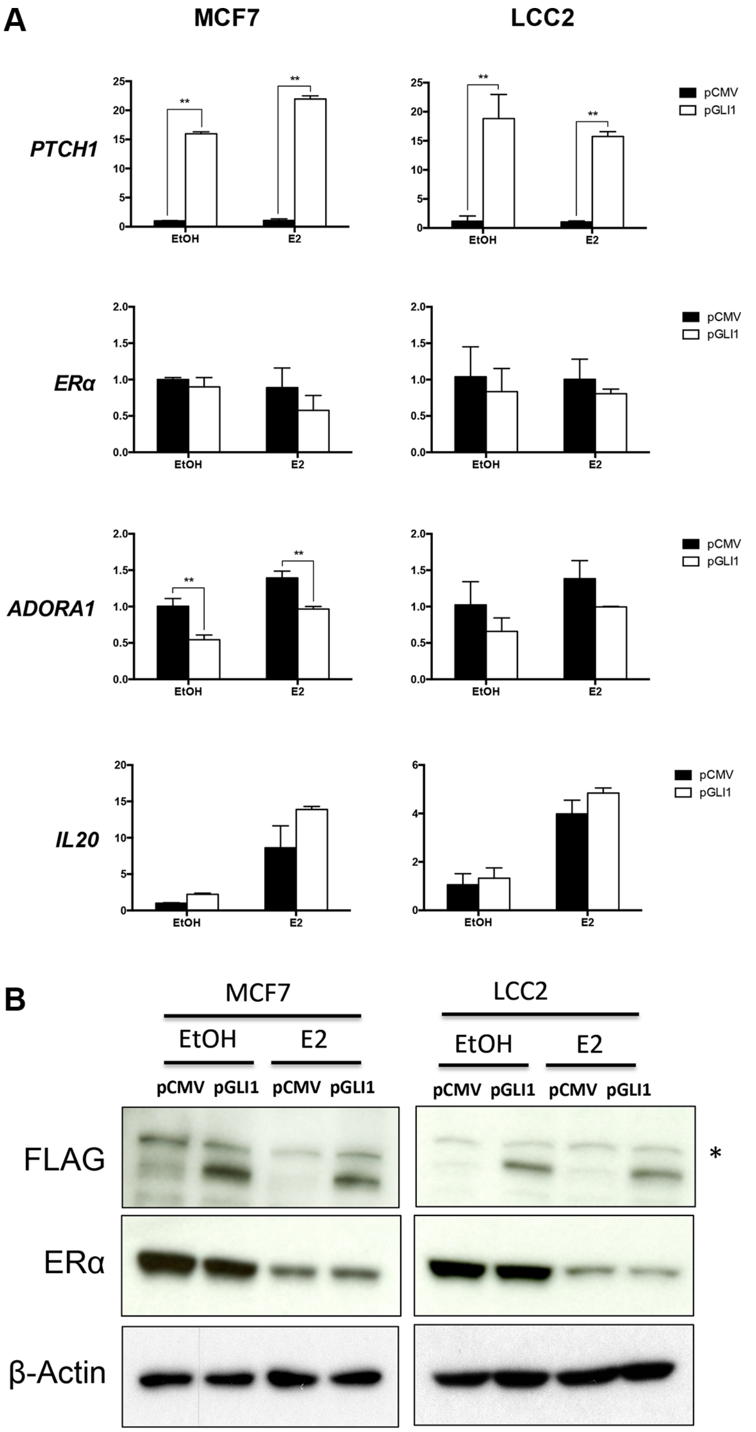

Supplementary Figure S5: GLI1 overexpression does not alter ERα signaling activity. (**A**) The expression of *PTCH1, ERα, ADORA1* and *IL20* in MCF7 and LCC2 cells transfected with FLAG-tagged GLI1 (pGLI1) and pCMV5 for 48 hours and treated with 10 nM E2 or ethanol (EtOH) for 3 hours in serum-deprived medium before harvesting, was determined by real-time PCR. Data are represented as relative expression ($2^{-\Delta\Delta Ct}$ values), calculated by subtracting the Ct value of the housekeeping gene *TBP* from the Ct value of the interrogated transcripts (ΔCt), and normalized to the ΔCt value obtained with the control pCMV in MCF7/LCC2 cells. Representative data from one of three independent experiments are shown. Error bars indicate the standard deviation. **, Statistical significant, $P < 0.01$, compared to control, calculated by the Student's *t*-test. (**B**) Protein levels of ERα in MCF7 and LCC2 cells transfected with FLAG-tagged GLI1 (pGLI1) and pCMV5 for 48 hours and treated with 10 nM E2 or ethanol (EtOH) for 3 hours in serum-deprived medium before harvesting, was determined by Western blot. Antibodies against FLAG (F7425, Sigma-Aldrich), ERα and β-Actin (control) were used. *, non-specific bands.

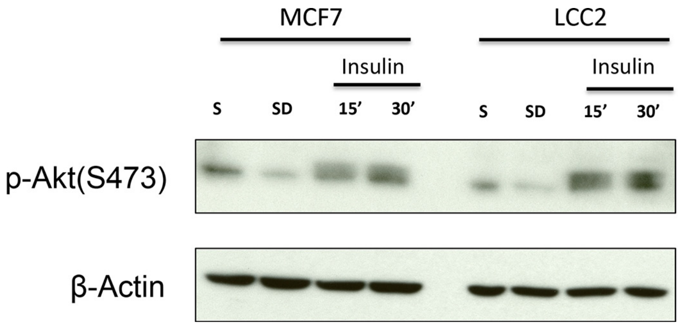

Supplementary Figure S6: Insulin treatment increases the activity of PI3K/Akt signaling in MCF7 and LCC2 cells. Cells were cultured in serum (S) or serum-deprived (SD) medium and stimulated with 100 nM insulin for 15 or 30 minutes. Total cell extracts were analyzed by Western blot using an antibody against phospho-Akt (Ser 473) (#9271, Cell Signaling Technology). β-Actin is used as a control.
